# Sex Differences in the Reinstatement of Methamphetamine Seeking after Forced Abstinence in Sprague-Dawley Rats

**DOI:** 10.3389/fpsyt.2015.00091

**Published:** 2015-07-06

**Authors:** Jana Ruda-Kucerova, Petra Amchova, Zuzana Babinska, Ladislav Dusek, Vincenzo Micale, Alexandra Sulcova

**Affiliations:** ^1^Experimental and Applied Neuropsychopharmacology Group, Central European Institute of Technology (CEITEC), Masaryk University, Brno, Czech Republic; ^2^Department of Pharmacology, Faculty of Medicine, Masaryk University, Brno, Czech Republic; ^3^Institute of Biostatistics and Analyses, Faculty of Medicine, Masaryk University, Brno, Czech Republic; ^4^Section of Pharmacology, Department of Biomedical and Biotechnological Sciences, School of Medicine, University of Catania, Catania, Italy

**Keywords:** methamphetamine, reinstatement of drug-seeking behavior, forced abstinence, sex/gender differences, Sprague-Dawley rats

## Abstract

Preventing relapse to drug abuse is one of the struggles faced by clinicians in order to treat patients with substance use disorders (DSM-5). There is a large body of clinical evidence suggesting differential characteristics of the disorder in men and women, which is in line with preclinical findings as well. The aim of this study was to assess differences in relapse-like behavior in methamphetamine (METH) seeking after a period of forced abstinence, which simulates the real clinical situation very well. Findings from such study might add new insights in gender differences in relapse mechanisms to previous studies, which employ a classical drug or cue-induced reinstatement procedure following the extinction training. Adult male and female Sprague-Dawley rats were used in IV self-administration procedure conducted in operant boxes using nose-poke operandi (Coulborn Instruments, USA). Active nose-poke resulted in activation of the infusion pump to deliver one intravenous infusion of METH (0.08 mg/kg). After baseline drug intake was established (maintenance phase), a period of forced abstinence was initiated and rats were kept singly in their home cages for 14 days. Finally, one reinstatement session in operant boxes was conducted. Females were found to self-administer significantly lower dose of METH. The relapse rate was assessed as a number of active nose-pokes during the reinstatement session, expressed as a percentage of active nose-poking during the maintenance phase. Females displayed approximately 300% of active nose-pokes compared to 50% in males. This indicates higher vulnerability to relapse of METH seeking behavior in female rats. This effect was detected in all females, independently of current phase of their estrous cycle. Therefore, this paradigm using operant drug self-administration and reinstatement of drug-seeking after forced abstinence model can be used for preclinical screening for potential new anti-relapse medications specific for women.

## Introduction

Methamphetamine (METH) addiction is a serious psychosocial problem, which leads to organic harm of the body as well as distortion of the normal functioning of affected people within the society and family. There is a large body of clinical evidence suggesting differential characteristics of the disorder in men and women. Despite the absolute number of female METH abusers being lower than the male ones, women usually appear more dependent, show higher escalation rates (1, 2) and most importantly tend to experience more frequent relapses (3, 4). These gender specific differences require specific treatment strategies for men and women (5–7). This particularly applies to relapse-prevention, which represents a key treatment challenge especially for women (8).

The preclinical approach to model drug addiction with the highest validity is usually considered as the operant drug self administration. To mimic relapse in this paradigm, a period of extinction procedure can be employed when the animal still has a regular access to the operant box but the drug delivered by infusion pump is replaced by vehicle. After certain number of sessions, the subject stops to respond to the active operandum (e.g., lever or nose-poke). After reaching a specific extinction criteria (number of active/inactive responses lower than a set number), one last session is conducted and the reinstatement of the drug-seeking behavior is primed by an environmental factor (stress, cues) or a drug dose. Such studies have repeatedly shown female rats to be more vulnerable to drug-primed relapse of METH seeking behavior at conditions of time limited sessions (2 h), which mimic rather consummatory behavior, as well as prolonged self-administration sessions. This is considered to provide a better model for loss of control over drug taking, leading to escalation of drug consumption (9) known from a clinical situation (3). Similarly, a higher relapse-like behavior was found in female rats after priming by conditioned cue and to even higher extent by METH dose (10). Earlier, analogous results were reported in studies with cocaine (11, 12) and fentanyl (13).

However, this paradigm does not mimic the human treatment very well, because the patient usually discontinues the drug abuse in the drug rehabilitation center and for some time does not have access to the drug-related environments. Therefore, a forced abstinence model was developed where the animal does not have access to the operant box and is kept in the home cage for some time (14–16); thus, the motivation of drug response behavior is not influenced by any training procedures.

Furthermore, many preclinical studies, which assess sex-dependent differences, isolate the hormonal effect either by ovariectomy and subsequent hormonal supplementation (17, 18) or by constant tracking of the estrous cycle phase (10, 19). These approaches already explained extensively the role of gonadal hormones in the reward processes showing enhancement of drug intake by estradiol (17, 18, 20–22) and attenuation of drug seeking by progesterone (4, 23). However, the possibilities of clinical applications of these findings are limited, so far only progesterone was tested as a treatment for nicotine relapse in women (24) and such treatment would have many undesirable side effects. Consequently, an ideal animal model with high face, construct, and predictive validity for testing new relapse-prevention treatments should not be based on hormonal levels only.

The intact animals (males and freely cycling females) showed no sex differences to effects of amphetamines in the animal model of conditioned place preference (CPP) (25, 26). Interestingly, CPP for METH did not occur in ovariectomized rats but developed in females treated with estradiol (27). Therefore, gender differences in the CPP paradigm might be biased by fluctuating hormonal levels in intact females. However, results supporting higher vulnerability to METH in intact female rats were reported too. Female rats displayed higher increase of locomotor activity, which lasted for longer time and had higher scores of stereotypies than male rats (28). These results indicate the sex differences may depend, besides hormonal influences, also on different pharmacokinetic processes in females (29).

Therefore, the aim of this study was to assess gender differences in all stages of operant IV self-administration of METH in male and female rats while the gonads of all animals were kept intact assuring physiological estrous cycle in females. We expected a higher variability in the female group, especially in the reinstatement of METH seeking behavior due to different hormonal stages. However, we hypothesized that this variability may be overpowered by all other significant gender differences. Furthermore, we assessed possible gender differences in acquisition and maintenance of food self-administration in order to compare the operant behavior toward natural reward (food) and the drug of abuse.

## Materials and Methods

### Animals

Eight-week-old male and female albino Sprague-Dawley rats weighing 175–200 g (females) and 200–225 g (males) at the beginning of the experiment were purchased from Charles River (Germany). The rats were housed individually in standard rat plastic cages, the experiments on males and females were performed separately, to assure the self-administration room is dedicated to one gender at a time only. Environmental conditions during the whole study were constant: relative humidity 50–60%, temperature 23 ± 1°C, inverted 12-h light–dark cycle (6 a.m. to 6 p.m. darkness). Food and water were available *ad libitum*. All experiments were conducted in accordance with all relevant laws and regulations of animal care and welfare. The experimental protocol was approved by the Animal Care Committee of the Masaryk University, Faculty of Medicine, Czech Republic, and carried out under the European Community guidelines for the use of experimental animals.

### Drugs and treatments

Methamphetamine from Sigma Chemical, Co., St Louis, MO, USA available in the operant cage for IV self-administration was 0.08 mg/kg per infusion with the maximum number of infusions obtainable in one session set to 50. The solutions were prepared for specific animals depending on their body weights rounded to the closest category of 250, 300, 350 g, etc. This paradigm is adapted from Emmett-Oglesby MW (Fort Worth, TX, USA) (30) and routinely used in our laboratory (17, 31–33).

### Locomotor activity test

After adaptation period at the beginning of the study basal behavioral profile was assessed in both males and females. In brightly lit room, rats were individually tested for locomotor activity using the Actitrack system (Panlab, Spain) as previously described (34, 35). Each Plexiglas arena (45 cm × 45 cm × 30 cm) was equipped with 2 frames equipped with photocells located one above another 2 and 12 cm above the cage floor. Each animal was placed in the center of arena and the spontaneous behavior was tracked for 10 min. During the test, the horizontal locomotor activity (the trajectory as calculated by the system from beam interruptions that occurred in the horizontal sensors) and vertical activity (number of rearing episodes breaking the photocell beams of the upper frame) were recorded. At the end of the session, animals were returned to their home cage and arenas were wiped with 1% acetic acid to avoid olfactory cues.

### Intravenous drug self-administration surgery

Animals were deeply anesthetized with i.p. injections of 50 mg/kg ketamine plus 8 mg/kg xylazine. Under aseptic conditions, a permanent intracardiac silastic catheter was implanted through the external jugular vein to the right atrium. The outer part of the catheter exited the skin in the midscapular area. After surgery, each animal was allowed for recovery, individually in its home cage with food and water freely available. Since the implantation, the catheters were flushed daily by heparinized cephazoline (Vulmizolin 1.0 g) solution followed by 0.1 ml of a heparinized (1%) sterile saline solution to prevent infection and occlusion of the catheter. During recovery, changes in general behavior and body weight were monitored. When a catheter was found to be blocked or damaged, the animal was excluded from the analysis. At the end of the study, there were *n* = 6 male and *n* = 6 female rats included to the analysis.

### Intravenous self-administration protocol

Methamphetamine self-administration was conducted as previously described (17, 32) in 10 standard experimental boxes (30 cm × 25 cm × 30 cm, Coulbourn Instruments, USA) using nose-poking as operandum under a FR-1 schedule of reinforcement, i.e., animal had to make 1 nose-poke on the active hole to obtain a single drug infusion. Each cage was provided with two nose-poke holes allocated on one side and programed by software Graphic State Notation 3.03 (Coulbourn Instruments, USA). Nose-pokes in the active hole led to the activation of the infusion pump and administration of a single infusion followed by a 10 s timeout, while nose-poke stimulation was recorded but not rewarded. The cage was illuminated by a house light during the session. The light was flashing when administering infusion and off during the time-out period. Self-administration sessions lasted 90 min and took place 7 days/week for 2 weeks in total between 8 a.m. and 3 p.m. during the dark period of the inverted light–dark cycle.

After 14 days of stable METH intake, the maintenance phase was terminated and rats were returned to their home cages for the 14 days of the forced abstinence period. On day 15, rats were placed into self-administration chambers for the last 90 min reinstatement session. The numbers of responses on the active drug-paired nose-poke and the inactive nose-poke were recorded but the drug was not delivered. Responses on the active nose-poke are considered to reflect the reinstatement of drug-seeking behavior, while responses on inactive nose-poke reflect non-specific locomotor and exploratory activity.

### Food self-administration protocol

Food self-administration was conducted in the same experimental boxes as METH study (Coulbourn Instruments, USA) in a separate batch of animals. Under the FR-1 schedule of reinforcement 1 nose-poke lead to activation of a feeder and delivery of a single palatable pellet (BioServ, sweet dustless rodent pellets, F0021-Purified Casein Based Formula – 45 mg). The cage was illuminated by a house light during the whole session. Self-administration sessions lasted 30 min during the dark period of the inverted light–dark cycle.

### Statistical data analysis

Primary data were summarized using arithmetic mean and SE of the mean estimate. Behavioral data were analyzed by *t*-test. IV METH self-administration data during the 14 days of maintenance were analyzed at individual days by *t*-test and at 5-day intervals by mixed ANOVA model with Greenhouse–Geisser correction. Acquisition of food self-administration was evaluated by comparison of mean day of reaching 70% preference of active nose poke by Mann–Whitney *U* test. Maintenance of food self-administration was analyzed at individual days by *t*-test. Statistical analyses were computed using SPSS 19.0.1 (IBM Corporation, 2010). A *p*-value <0.05 was recognized as boundary of statistical significance in all applied tests.

## Results

### Basal locomotor characteristics

**Figure 1:**
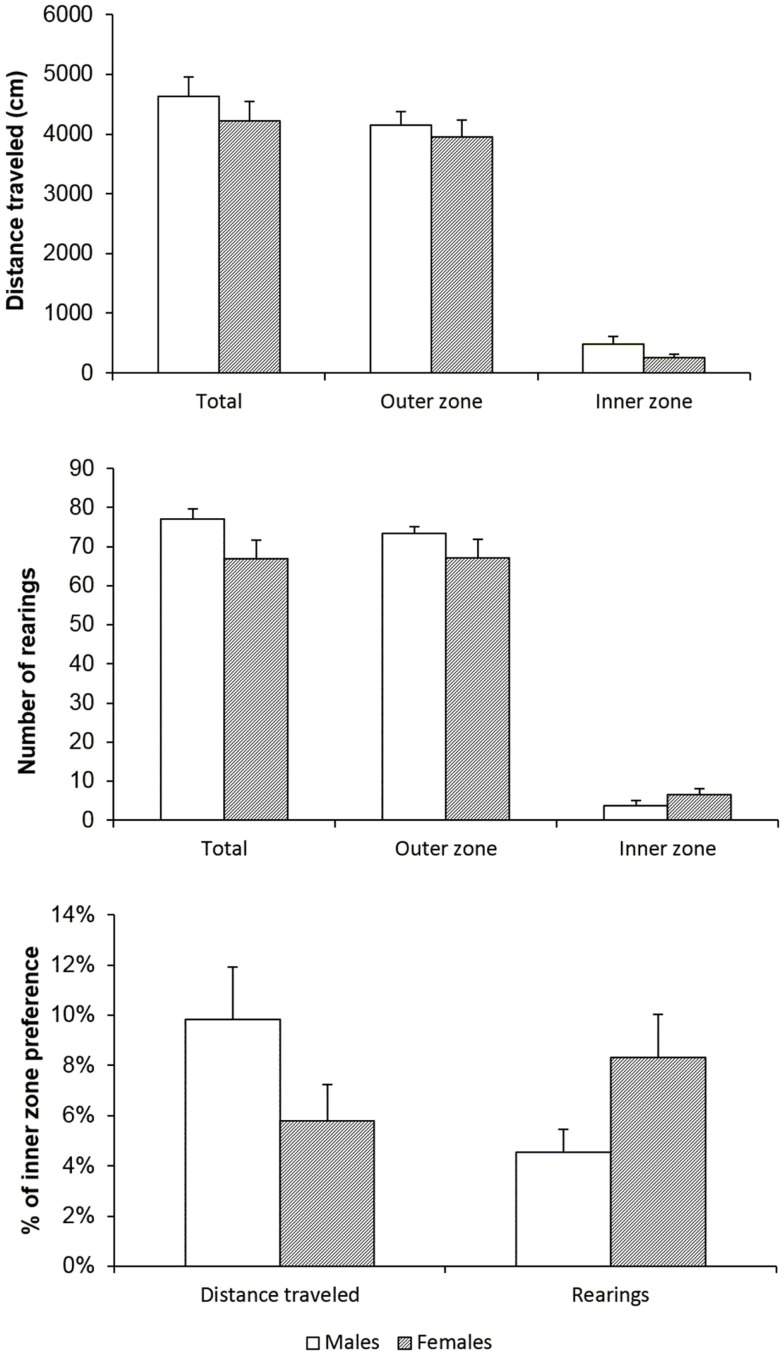
**Male and female rats have the same basal behavioral profile**. Total distance traveled (in centimeters), number of rearing episodes (vertical activity), and % of preference of the central part of the arena did not reveal any gender difference. Data are shown as means (±SEM), *t*-test, n.s.

Before starting the IV self-administration protocol, basal locomotor and exploratory activity was assessed in both males and females to exclude the possibility that these characteristics would lead to different drug taking behavior. Horizontal and vertical locomotor activity was measured and a proportion of each in the inner zone of the arena was calculated in order to evaluate differences in the status of anxiety in male and female rats. Figure 1 illustrates the results on total distance traveled, vertical activity (number of rearing episodes), and inner part of arena preference. There were no basal behavioral differences between the sexes, which could contribute to dissimilar behavior in the operant cage. As expected, both sexes avoided the central part of the arena, which represents normal rodent behavior and neither one shows highly anxiogenic behavior or locomotor hyper- or hypo-activity.

### Acquisition and maintenance of methamphetamine self-administration in male and female rats

**Figure 2:**
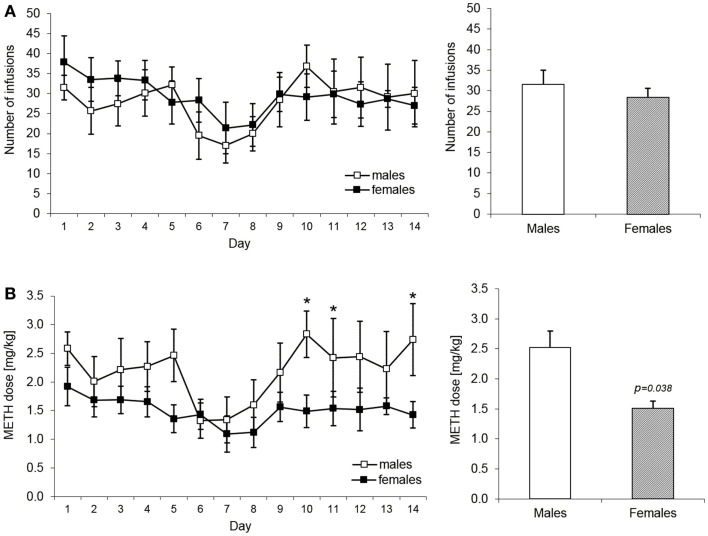
**Acquisition and maintenance of methamphetamine intake in male and female rats**. The **(A)** part shows number of infusions expressed as daily means over the 14 days of acquisition and maintenance of the METH IV self-administration. The bar graph depicts the mean number of infusions over the whole 14 days period. There were no statistically significant differences in this measure (mixed ANOVA model). The **(B)** part shows in an analogical way the mean dose in milligram per kilogram of METH self-administered by male (*n* = 6) and female (*n* = 6) rats. The groups start to differ significantly from the day 10 with *t*-test results: day 10 (*p* = 0.021), day 11 (*p* = 0.049), and day 14 (*p* = 0.048). The bar graph shows the mean number of infusions over the last 5 days of the maintenance period (day 10–14) when the drug intake started to be significantly higher in male rats (*p* = 0.038, mixed ANOVA model).

The acquisition and maintenance of METH taking behavior were assessed, first, in terms of mean number of infusions self-administered per session and, second, by the mean METH dose per session in milligram per kilogram. Figure 2A shows number of infusions obtained per daily session and mean number of infusions during the entire acquisition phase in male and female rats during the acquisition phase of METH self-administration training. ANOVA revealed no significant effects over the whole period. However, when the number of infusions was converted to a METH dose per kilogram of body weight, males were found to self-administer higher dose at the end of the acquisition phase as compared to females. More specifically, as depicted in the Figure 2B, mean METH intake during the last 5 days of training was significantly higher in males than in females, i.e., 2.5 and 1.5 mg/kg, respectively (mixed ANOVA model: *p* = 0.038).

### Reinstatement of methamphetamine self-administration in male and female rats

**Figure 3:**
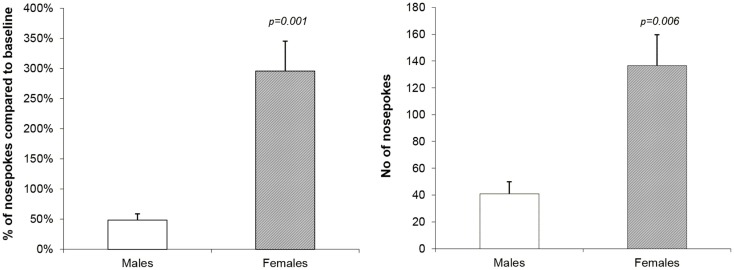
**Reinstatement of methamphetamine seeking behavior in male and female rats**. The graphs show a percent of mean basal nose-poking (14 days of acquisition and maintenance) and number of nose-pokes in the reinstatement session in male and female rats. There was a statistically significant difference between the sexes in both measures: male rats showed mean % of responding 48.3% and females 295.7% (mixed ANOVA model, *p* = 0.001). The apparent difference between the sexes is further confirmed by behavioral activity reflected in a mean number of nose-pokes: 41.0 in males and 136.5 in females (mixed ANOVA model, *p* = 0.006).

After the 2-week-long period of forced abstinence one last reinstatement session was performed with no drug availability. The only measure of the drug-seeking behavior is the number of active operandum responses. This number was converted to a percent of mean basal nose-poking (14 days of acquisition and maintenance). There was a massive difference between the sexes recorded: male rats showed mean percent of responding 48.3% whereas females showed 295.7% (mixed ANOVA model, *p* = 0.001). Results are reported on the Figure 3.

### Acquisition of food self-administration in male and female rats

**Figure 4:**
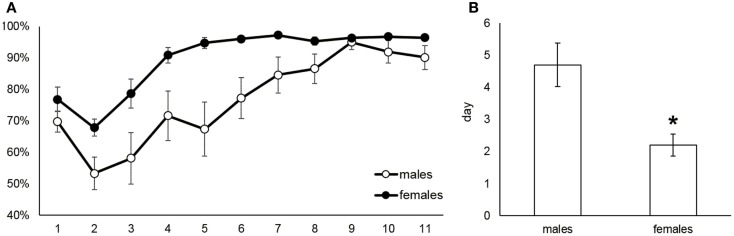
**Acquisition of food self-administration behavior in male and female rats**. The **(A)** part shows the course of the active nose poke preference development during acquisition and maintenance of the food self-administration. The bar graph **(B)** depicts the mean day the animals reached and kept 70% preference of the active operandum: 4.7 in males and 2.2 in females (Mann–Whitney *U* test, *p* = 0.014).

**Figure 5:**
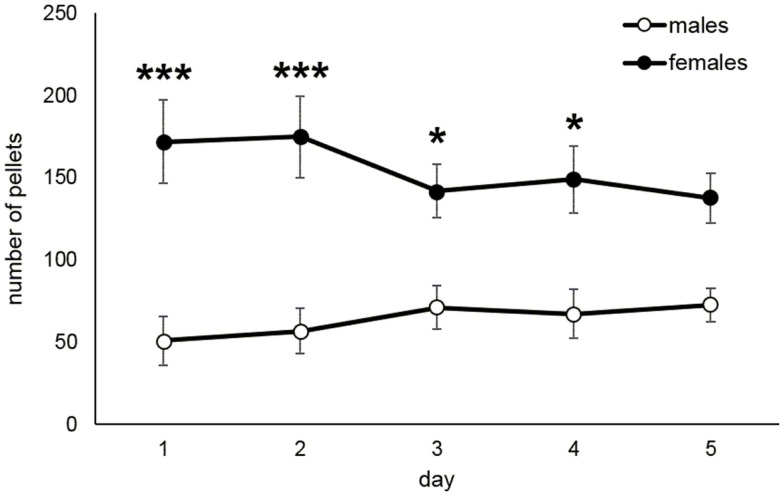
**Maintenance of food self-administration in male and female rats**. The graph shows pellet intake in female rats as compared to males (*t*-test, *p ≤ 0.05, ***p ≤ 0.001).

The acquisition of food taking behavior (sweet pellets) was assessed in terms of day when the animals started to prefer the active nose-poke more than 70%. Figure 4A shows the development of active nose poke preference (%) over all sessions in male and female rats. Figure 4B reports the mean day for reaching 70% preference of the active operandum, which was 4.7 in males and 2.2 in females (Mann–Whitney *U* test, *p* = 0.014). The maintenance phase of the food self-administration was evaluated as a mean number of self-administered pellets during the last 5 days when the intake was stable. Figure 5 depicts the significantly higher pellet intake in female rats as compared to males (138–175 and 51–73, respectively, *p* ≤ 0.05).

## Discussion

Findings of the present study demonstrated that male and female rats had equal basal locomotor and exploratory activity. Thus, differences in operant IV self-administration cannot be accounted for differences in locomotor activity. Furthermore, the food self-administration has shown a very different dynamics than the METH study, suggesting higher motivation to obtain natural reward (sweet pellet) in females, which learned the operant procedure faster (acquisition) and self-administered approximately three times more pellets than males. This behavior toward natural reward is very different from METH-related operant behavior, which rules out the possibility of general gender specific difference in the reward processes.

During the maintenance phase of the METH self-administration, female rats were found to self-administer the same number of infusion, but their METH intake in terms of dose per kilogram of body weight was found lower. This measure is not widely used among the self-administration studies, usually only the numbers of nose pokes (or lever presses) and infusions are reported. However, we propose this measure to be considered as highly valid for several reasons. Despite the solution of the drug being available in the operant box matches the body weight of the particular animal, the solutions are prepared for certain body weight category, e.g., solution for animal weighting 300 g can be used for rats reaching approximately 280–320 g (this fact is usually not described exactly in the Section “Materials and Methods” of the papers). This discrepancy, aggravated by the fact that the body weight of the animal changes over the course of the experiment, could be a source of significant differences in the dose taken even at conditions of the same number of infusions. This is a confounding factor, which complicates the comparison of findings from different laboratories. Furthermore, this approach should be used when the number of behavioral responses (nose pokes or lever presses) does not match the number of infusions delivered. This is always the case when the system uses nose poke operandi (and in some cases levers which do not retract after infusion delivery).

Previous studies have shown that female rats to be more vulnerable to behavioral effects of psychomotor stimulants including cocaine (36–38) and, in particular, amphetamines (including METH), which elicited a higher increase of locomotion in females than males or reach the same behavior profile at lower dose (25, 28, 39, 40). Other studies have repeatedly shown that females with intact gonads tend to develop readily behavioral sensitization to psychostimulant drugs after repeated treatment (41–43). Furthermore, there is new evidence of specific pharmacokinetic differences in METH self-administration studies, where males were shown to have lower area under curve (AUC) of METH probably due to rapid drug elimination (29). The apparent higher efficacy of the amphetamines found in this and previously mentioned studies in female rats could be explained by the pharmacokinetic differences.

Similarly, in clinical studies, there has been shown that men are more sensitive to the reinforcing effects of a high dose of d-amphetamine than women, who respond rather to low doses at a random phase of the menstrual cycle (44). This is consistent with our data, which showed that males developed higher stable intake of METH than females (2.5 and 1.5 mg/kg daily, respectively). Furthermore, women were shown to experience greater increases in diastolic pressure and nausea than men at the same doses while the ability to discriminate d-amphetamine was equal in both sexes (45). These lines of evidence further support translational validity of our finding of lower METH intake in female rats.

However, in fixed ratio self-administration paradigms, the reports on gender differences in the maintenance phase are numerous and quite contradictory in both clinical (45, 46) and preclinical studies, showing both higher and lower drug intake in female subjects (21, 47).

Progressive ratio IV self-administration paradigm or prolonged access to the drug might be better tools to unravel gender differences as these may be linked to appetitive behavior (21). Female rats have been repeatedly shown to achieve higher breaking points in METH self-administration study suggesting higher motivation to obtain the drug (10, 48). This is consistent with the robust gender difference in the reinstatement found in this study, where the motivation of animals for the drug-seeking was not abolished by extinction training. At this point, active responses to the operandum are the only measure to report because the session is performed without delivering the drug. We found a highly significant difference in the percent of mean basal nose-poking, as well as in the absolute number of active operant responses. The enhancing effect of estradiol and attenuating effects of progesterone on psychostimulant (d-amphetamine, METH, cocaine) intake in female gender is repeatedly and consistently reported in both clinical (49–52) and preclinical studies (17, 20–22). Therefore, the higher variability in the reinstatement operant responding in the female group detected in this study probably originated from different hormonal stage. This conclusion can be supported by an earlier study, which employed the extinction and both drug- and cue-primed reinstatement, where females were found more vulnerable in both reinstatement procedures and also exhibited higher variability than males. Interestingly, the numbers of lever presses in the conditioned cue-primed reinstatement session were approximately 40 in males and 120 in females (10). These absolute numbers are similar to those reported in the present study: 41 and 136, respectively. Therefore, this effect seems to be well reproducible and strain independent (Long-Evans vs. Sprague-Dawley rats).

The forced abstinence model was proposed as a potentially better tool to model a spontaneous relapse in rodents (15, 53). To our knowledge, this is the first report of gender differences in the paradigm of reinstatement after forced abstinence. Extinction-based approach to study relapse-like behavior phase in the preclinical setting show contradictory results – females appear to meet the extinction criteria later than males (11), but negative results have been reported as well (54). Both studies were conducted with cocaine.

Taken together, this study reports lower consummatory METH intake during maintenance phase of the self-administration together with higher vulnerability to the reinstatement of METH seeking behavior in female rats after forced abstinence. These effects seem to be robust enough, thus relatively independent on the current hormonal level. Therefore, we propose this paradigm for preclinical screening for potential new medications specific for women. However, the main limitation for the translation of these results to human medicine is the absence of psychosocial aspects, which are impossible to reflect in animal studies.

## Conflict of Interest Statement

The authors declare that the research was conducted in the absence of any commercial or financial relationships that could be construed as a potential conflict of interest.
